# Unhealthy Days and Quality of Life in Irish Patients with Diabetes

**DOI:** 10.1371/journal.pone.0081102

**Published:** 2013-12-13

**Authors:** Emma Louise Clifford, Margaret M. Collins, Claire M. Buckley, Anthony P. Fitzgerald, Ivan J. Perry

**Affiliations:** 1 Department of Nutrition & Dietetics, South Infirmary Victoria University Hospital, Cork, Ireland; 2 University of California Co-Operative Extension, Sonora, California, United States of America; 3 Department of Epidemiology & Public Health, University College Cork, Cork, Ireland; 4 Department of Statistics, University College Cork, Cork, Ireland; University of North Carolina at Chapel Hill, United States of America

## Abstract

**Objectives:**

To study the determinants of health-related quality of life (HRQoL) in Irish patients with diabetes using the Centres for Disease Controls' (CDC's) ‘Unhealthy Days’ summary measure and to assesses the agreement between this generic HRQoL measure and the disease-specific Audit of Diabetes Dependant Quality of Life (ADDQoL) measure.

**Research Design and Methods:**

Data were analysed from the Diabetes Quality of Life Study, a cross-sectional study of 1,456 people with diabetes in Ireland (71% response rate). Unhealthy days were assessed using the CDC's ‘Unhealthy days’ summary measure. Quality of life (QoL) was also assessed using the ADDQoL measure. Analyses were conducted primarily using logistic regression. The agreement between the two QoL instruments was measured using the kappa co-efficient.

**Results:**

Participants reported a median of 2 unhealthy days per month. In multivariate analyses, female gender (P = 0.001), insulin use (P = 0.030), diabetes complications (P = <0.001) were significantly associated with more unhealthy days. Older patients had fewer unhealthy days per month (P = 0.003). Agreement between the two measures of QoL (unhealthy days measure and ADDQoL) was poor, Kappa = 0.234

**Conclusions:**

The findings highlight the determinants of HRQoL in patients with diabetes using a generic HRQoL summary measure. The ‘Unhealthy Days’ and the ADDQoL have poor agreement, therefore the ‘Unhealthy Days’ summary measure may be assessing a different construct. Nonetheless, this study demonstrates that the generic ‘Unhealthy Days’ summary measure can be used to detect determinants of HRQoL in patients with diabetes.

## Introduction

The prevalence of diabetes is rising globally, with the number of people with diabetes increasing from 153 (127–182) million in 1980, to 347 (314–382) million in 2008 [1]. Improving quality of life (QoL) is one of the major goals in the St. Vincent Declaration for people with diabetes [2]. Patients with poor quality of life (QoL) are less likely to comply with their dietary regime, be physically active and manage diabetes self-care. There have been numerous instruments developed over the years to measure QoL and health related quality of life (HRQoL) leading to on-going debate on whether generic or disease-specific measures have greater relative merit. Generic instruments, such as the Short-Form-36 (SF-36) [3]–[6], EuroQoL (EQ-5D) [5], [7]–[8] and the Centres of Disease Control's (CDC's) HRQoL-4 measures [9]–[11] have been used in the literature on patients with diabetes. Examples of diabetes disease-specific instruments most widely used are the Audit of Diabetes Dependant Quality of Life (ADDQoL) [3]–[5], Diabetes Quality of Life Questionnaire (DQOL) [6], and the Problem Areas in Diabetes Scale (PAID) [6].

Several studies have identified predictors of HRQoL in diabetes using both generic and disease-specific instruments [3], [5]–[6], [8]–[15]. Overall, the most significant determinants of poor HRQoL are insulin use [5], [8], [10], obesity [3], [5], [8] and diabetes related complications, [3], [8], [13]–[14], [16]. Specifically, studies using the generic CDC's HRQoL-4 measures have shown that having diabetes is associated with poor physical health [11], [17] and poor mental health [11]. Cross-sectional studies using the ADDQoL have also shown that patients with diabetes have poor QoL [3]–[5], especially those that are obese [3], [8], those with type 1 diabetes [4], those using insulin [3]–[4], [8], [10], and those with diabetes related complications [3]–[4], [8]. Notably, QoL has been found to be better in older people using disease specific instruments (ADDQoL, DQOL, PAID, ADS) [4], [5], [6]. A substantial body of literature has focused on comparing generic and disease-specific measures [3], [7], [12], [18]–[21]. Disease-specific instruments focus on a population with a specific disease e.g. diabetes, asthma or rhinitis and their advantage is that they are more responsive to treatment related change [22]–[24]. Generic instruments are designed to investigate aspects of health that are of universal importance [21] and are applicable to both healthy people and those with disease and are therefore more generalizable [20]. Rubin & Peyrot suggest that using both generic and disease-specific instruments provides a more comprehensive assessment of HRQoL in patients with diabetes [14]. The generic CDC's healthy days measures are a set of measures of health status and activity limitation [25]. The HRQoL-4 measures are four core measures of the healthy days set and are widely used in the U.S. [25]. The ‘unhealthy days’ summary measure is based on the second and third questions of the HRQoL-4 and it estimates the overall number of recent days when physical or mental health was not good [25]. Experience in using this instrument in patients with diabetes is limited and few published studies have specifically examined determinants of HRQoL in diabetes using these measures. Moreover, studies using the CDC's ‘unhealthy days’ summary measure as an instrument to measure HRQoL is very limited.

Our aim was to study the determinants of HRQoL in Irish patients with diabetes using CDC's ’unhealthy days’ summary measure. We also address the level of agreement between this generic HRQoL measure and the ADDQoL, an established disease-specific instrument in the measurement of QoL in these patients.

## Research Design and Methods

Data were collected from the Diabetes Quality of Life Study, which is a cross-sectional study involving 2,049 Irish people aged 20 to 75 years with a confirmed diagnosis of type 1 or 2 diabetes (71% response rate) [4], [26]. Patients were recruited from those attending three different models of diabetes care in Ireland. The self-completed questionnaire addressed standard demographic, social and clinical factors including sex, age, educational and marital status, type of diabetes, insulin use, body mass index (BMI) and diabetes related complications. Details of the methodology and recruitment are available elsewhere [4], [26]–[27].

### Ethics Statement

The study was approved by the Clinical Research Ethics Committee of the Cork Teaching Hospitals and the Irish College of General Practitioners (ICGP) Ethics Committees. All patients gave written informed consent.

### Generic HRQoL instrument: Centres for Disease Controls' ‘Unhealthy Days’ Summary Measure [28]

HRQoL was assessed using the ‘unhealthy days’ summary measure from the CDC's HRQoL-4 core Healthy Days measures [28]. These four measures are the briefest set of validated generic HRQoL measures, based on understandable and clear definition of HRQoL [25], [28]–[29]. They were derived from the original version of the Medical Outcomes Study 36-item short-from survey (SF-36) instrument [9], [28]–[30] and have been validated in both healthy and disabled populations with acceptable criterion validity and reliability comparable with multiple item SF-36 subscales [9], [25], [28]–[30]. They are used yearly in the Behavioural Risk Factor Surveillance System (BRFSS) telephone survey in the U.S. [28]. Based on average times, the HRQoL-4 takes about 1.0 minute to administer via telephone [28]. The four questions include: 1) self-rated health, (poor, fair, good, very good, excellent) 2) number of recent days when physical health was not good, (0–30days) 3) number of recent days when mental health was not good (0–30 days) and 4) number of recent days that poor physical or mental health kept you from doing your usual work or recreational activities (0–30 days). Recent is defined as during the previous 30 days [28]. Unhealthy days are an estimate of the overall number of days during the previous 30 days when the respondent felt that they had poor physical or mental health. For the purpose of this research, we focused on total unhealthy days (unhealthy days summary measure), which is the sum of the number of days of poor physical health (question 2) and poor mental health (question 3), with a logical maximum of 30 unhealthy days per month [28]. The unhealthy day's summary measure provides a simple assessment of perceived physical and mental health over time, with good concurrent and acceptable criterion validity [25], [28]–[30]. The more unhealthy days reported per month, the poorer the HRQoL.

### Disease-specific instrument: Audit of Diabetes Dependant Quality of Life (ADDQoL) [31]

QoL was also measured using the ADDQoL 18 item instrument which is an established disease-specific instrument measuring the impact of diabetes upon the individual [31]. The ADDQoL is a well-recognised QoL measurement tool with good psychometric properties and it is now translated into twenty languages [32], [33]. It measures the impact of diabetes on 18 items representing domains of life: work, family, sex and social life, finances, physical appearance, physical activities, travel, self-confidence, motivation, dependence, living conditions, society reaction, future, freedom to eat and drink and enjoyment of food. Scores are on a scale of +9 to −9, the more negative the score, the greater the impact of diabetes on QoL, therefore poorer QoL.

### Statistical Analyses

The principle analyses of the data focused on the association between the unhealthy day's summary measure and socio-demographic and clinical variables in patients with diabetes. We dichotomised the unhealthy days (days 0 to 30), and used the reporting of no unhealthy days *(none)* per month vs. reporting of *one or more unhealthy days* (1–30) per month. Non-parametric Mann-Whitney U test and the Kruskal-Wallis test were used to compare the median number of unhealthy days in different patient subgroups. Binary logistic regression analysis was used to assess whether the reporting of unhealthy days was independently associated with any of the socio-demographic or clinical variables. The recoded unhealthy day's measure (*none vs*. *one or more*) was used as the dependant variable. The prevalence odds ratio (OR's) and 95% Confidence Intervals were estimated for the reporting of unhealthy days. It was first run adjusted for gender and age only, then fitted with the all categorical variables and re-run adjusting for confounders. The relationship between the ADDQoL score (−9 to +9) and the unhealthy days summary measure (0–30 days) was explored using Spearman's Rank Order Correlation. Agreement between these measures of QoL was also assessed using the Kappa statistic. The data were dichotomised prior to these analyses: ADDQoL score in the upper quartile vs. quartiles 1–3 [4], and the unhealthy days: reporting *of none vs. one or more*. Kappa coefficient assesses inter-rater agreement between two instruments on a scale of 0 to 1 [34]. Data analyses were conducted using SPSS version 18.0 for Windows.

## Results

### Participants

The overall response rate was 71% (N = 1456). The sample was composed of 42.1% females. Over half (53.6%) of participants were in the 60+ year's age category. Almost 80% (n = 1160) had type 2 diabetes and 62.2% (n = 721) with of those with type 2 diabetes were aged 60+ years. Only 23% of participants aged 60+ years had type 1 diabetes. Of those with type 2 diabetes, only 17.8% (n = 179) were using insulin. Almost a third (32.3%) of subjects had a BMI of >30 kg/m2. We found that Irish patients with diabetes reported a median of 2 (IQR 0–10) unhealthy days in the past month and diabetes impacts negatively on QoL, with an ADDQoL score of −1.7 (IQR −3.56 to −0.65). Almost half of participants, reported no unhealthy days per month (n = 624, 49%), with 51% (n = 649) reporting one or more unhealthy days per month. Table 1 summarises the findings from univariate analysis of unhealthy days by age, gender, socio-demographic and clinical variables. Older age (P = 0.001) and married status (0.047) were significantly associated with fewer unhealthy days in the past month. Female gender (P = <0.001) was associated with an increased number of unhealthy days. There was no significant difference in age between males (median 60 years) and females (median 61 years) (P = 0.57). Insulin use was associated with having more unhealthy days per month (P = 0.035). Having diabetes complications increased the number of unhealthy days per month (P = <0.001). Almost half of females (n = 295, 48.2%) had two or more complications but there was no significant difference in the number of complications between males and females (P = 0.44). There was no significant difference when comparing mean BMI of males and females (P = 0.59). Type of diabetes and educational status were not significantly associated with reporting unhealthy days and there was no significant association between gender and educational status (P = 0.12)

**Table 1:** Distribution of Unhealthy Days by socio-demographic and clinical variables (N = 1456).

Variable		*N (%)	Median (IQR) (unhealthy days summary index)	**p-value
**Gender:**	Male	842 (57.9)	0 (0–9)	<0.001
	Female	612 (42.1)	3 (0–14)	
**Age:**	20–39 years	121 (8.4)	3 (0–9)	0.001
	40–59 years	546 (38)	2 (0–13)	
	60+ years	768 (53.6)	0 (0–10)	
**Type:**	Type 1	296 (20.3)	2 (0–8.5)	0.87
	Type 2	1160 (79.9)	1 (0–11)	
**Education:**	Primary	601 (41.3)	1 (0–10)	0.62
	Lower Secondary	339 (23.3)	2 (0–9.5)	
	Completed Secondary	227 (16.6)	1 (0–7)	
	Tertiary	180 (12.4)	2 (0–10)	
	Unknown	63 (4.3)	1 (0–23)	
**Marital Status:**	Married	966 (66.3)	1 (0–10)	0.05
	Unmarried	477 (32.8)	2 (0–15)	
**Insulin Use:**	Yes	462 (31.7)	2 (0–10)	0.04
	No	828 (56.9)	0 (0–10)	
**Diabetes Complications:**	None	408 (28)	0 (0–5)	<0.001
	One	342 (23.5)	1 (0–8)	
	Two or more	706 (48.5)	4 (0–16.5)	
**BMI:**	<24.9 kg/m2	371 (26.2)	2 (0–10)	<0.001
	25–29.9 kg/m2	572 (40.4)	0 (0–7.5)	
	>30 kg/m2	474 (33.5)	3 (0–14)	

Number (N) for individual variables will vary because of missing values.

P value obtained with Mann-Whitney U test or Kruskal Wallis test as appropriate.

= body mass index. BMI

We found significant difference in mean BMI between those reporting no unhealthy days per month (27.4 kg/m2) and those reporting one or more unhealthy days per month (28.4 kg/m2) (P = 0.02). The mean difference was 0.96 kg/m2, [CI 0.14–1.75]. A Kruskal-Wallis was conducted to explore the impact of BMI on the number of reported unhealthy days. We dichotomised BMI again into three categories: healthy (BMI <24.9 kg/m2), overweight (BMI 25–29.9 kg/m2) and obese (>30 kg/m2). There was a statistically significant difference in reporting of unhealthy days across the three categories (P = <0.001). The median number of unhealthy days reported was 2 days for BMI <24.9 kg/m2, 0 days for BMI 25–29.9 kg/m2 and 3 days for those with a BMI of 30+kg/m2, and the difference was statistically significant across all three groups (P = <0.001).

### Logistic Regression

Table 2 summarises the findings from logistic regression analyses on the determinants of unhealthy days. After adjustment for age, females were more likely to report one or more unhealthy days [OR 0.74, CI 0.56–0.88, p = 0.002]. After adjustment for gender, participants aged 60+years were less likely to report unhealthy days [OR 0.64, CI 0.43–0.95, p = 0.028]. After adjustment for age and gender only; types of diabetes, educational status, marital status, insulin use or BMI were not significantly associated with unhealthy days. Those with two or more diabetes complications were more likely to report unhealthy days [OR 2.71, CI 2.04–3.60, p = <0.001]. In multivariate analyses, female gender continued to be predictive for reporting one or more unhealthy days [OR 0.66, CI 0.51–0.84, p = 0.001]. The chances of reporting one or more unhealthy days decreased with older age [OR 0.45, CI 0.26–0.77, p = 0.004]. Those using insulin were more likely to report unhealthy days [OR 1.52, CI 1.04–2.22, p = 0.030]. The chances of reporting unhealthy days increased almost threefold in those with two or more complications [OR 2.75, CI 1.99–3.77, p = <0.001]. Those with a BMI of 25–29.9kg/m2 were less likely to report unhealthy days [OR 0.71, CI 0.52–0.99, p = 0.04]. Types of diabetes, educational and marital status were not significantly associated with unhealthy days.

**Table 2:** Determinants of Unhealthy Days (one or more unhealthy days in the past month) in patients with types 1 and 2 diabetes.

Determinants of Unhealthy Days	OR*	CI (95%)	p-value	OR**	CI (95%)	p-value
**Sex**: Male vs. *Female*	0.74	0.56–0.88	0.002	0.66	0.51–0.84	0.001
**Age**: 40–59 yrs vs. *20–39 yrs*	0.96	0.63–1.45	0.84	0.79	0.48–1.29	0.35
**Age**: 60+ yrs vs. 20–*39 yrs*	0.64	0.43–0.95	0.03	0.45	0.26–0.77	0.004
**Type**: Type 2 vs. *Type 1*	0.94	0.66–1.24	0.53	0.71	0.44–1.14	0.16
**Education**: lower secondary vs. *primary*	0.91	0.68–1.22	0.52	1.07	0.77–1.50	0.69
**Education**: completed secondary vs. *primary*	0.84	0.60–1.17	0.31	1.04	0.71–1.51	0.84
**Education**: tertiary vs. *primary*	1.14	0.79–1.64	0.49	1.37	0.91–2.16	0.13
**Education**: unknown vs. *primary*	0.98	0.53–1.78	0.94	1.07	0.53–2.15	0.85
**Marital status**: married vs. *unmarried*	1.23	0.96–1.55	0.93	1.12	0.93–1.60	0.15
**Insulin use**: insulin vs. *no insulin use*	1.27	0.98–1.65	0.07	1.52	1.04–2.22	0.03
**Diabetes Complications**: One vs. *none*	1.67	1.2–2.30	0.002	1.77	1.24–2.53	0.002
**Diabetes Complications**: two or more vs. *none*	2.71	2.04–3.60	<0.001	2.75	1.99–3.77	<0.001
**BMI**: 25–29.9 kg/m2 vs. *<24.9 kg/m2*	0.78	0.58–1.03	0.08	0.71	0.52–0.99	0.04
**BMI**: >30 kg/m2 vs. *<24.9 kg/m2*	1.28	0.95–1.72	0.11	1.12	0.76–1.58	0.56

Reference group in Italics.

Logistic regression model for each variable, adjusted for age and sex only.

Logistic regression model adjusted for age, sex and all other variables in the table.

= body mass index. BMI

### Agreement between measures

**Figure 1:**
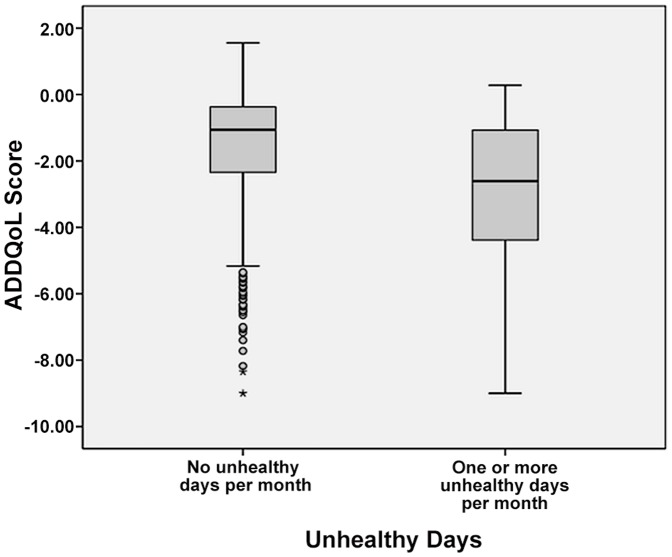
Distribution of ADDQoL score by unhealthy days groups. Figure 1 shows the variability of the ADDQoL score within the dichotomised unhealthy day's groups. While the distribution of the ADDQoL scores was shifted to lower levels in those with one or more unhealthy days relative to those with none, there was significant overlap between the two groups.

The relationship was explored using Spearman's Rank Order Correlation. There was a medium, negative correlation between the ADDQoL scores and the number of unhealthy days (0–30), r = −0.368, P = <0.001, with a higher number of unhealthy days associated with a lower ADDQoL score. There was ‘poor agreement’ between the ADDQoL scores (4^th^ versus 1^st^ to 3^rd^ quartile) and unhealthy days (*none vs. one or more*), with a kappa coefficient of 0.234. Figure 1 shows the variability of the ADDQoL score within the dichotomised unhealthy day's groups. While the distribution of the ADDQoL scores was shifted to lower levels in those with one or more unhealthy days relative to those with none, there was significant overlap between the two groups.

## Discussion

This study provides a better understanding on the determinants of HRQoL in diabetes and is the one of the first published studies to use the generic CDC's unhealthy day's summary measure on patients with diabetes. We found female gender, insulin use and diabetes complications to be significantly associated with poor HRQoL. We found that older patients to have significantly better HRQoL. Surprisingly, we found that being overweight was significantly associated with reporting no unhealthy days when compared to being a healthy weight or obese. Additionally, we tested the hypothesis that there would be an association between the unhealthy day's summary measure and the disease-specific ADDQoL instrument. We found that the shorter unhealthy day's summary measure could not be used as an independent predictor of ADDQoL scores; however it is sensitive enough to detect the determinants of HRQoL in patients with diabetes.

Collins et al, in the same study population, found individuals who were 60+ years of age reported higher ADDQoL scores than those who were younger (20–59 years). Insulin use and diabetes complications were significantly associated with lower ADDQoL scores, similar to our findings on the same patient group [4].

The findings from this study can be compared with results from other countries. We found that Irish patients with diabetes reported a median of 2 unhealthy days in the past month. Prevalence estimates of mean unhealthy days in the U.S. general population in 2010 were 6.2 unhealthy days per month, using the BRFSS trend data [35]. Brown et al., examined adults with diabetes using data from the 2001 BRFSS in the U.S, and found the mean number of physically or mentally unhealthy days were 9.7 [10]. Campbell et al. used data from the 2005 BRFSS, and having diabetes was associated with an average of 9 days of poor physical health and 4.6 days of poor mental health in the previous month [10]. The disparity in reporting of unhealthy days between Ireland and the U.S. is difficult to understand and unfortunately data on unhealthy days in the general Irish population are unavailable. However, a recent survey carried out by the Organisation for Economic Co-operation and Development (OECD) across 34 countries measuring well-being, ranks Ireland among the top ten countries with better overall well-being using a ‘better life index’[36]. This may explain our findings on the reporting low unhealthy days.

Consistent with previous research using generic instruments, we found female gender to be a significant predictor for reporting unhealthy days, therefore poor HRQoL [8], [15]. This may be due to women's role in society, with multiple responsibilities, juggling work-life, family-life and their diabetes regimen. We found those in the 60+ age group to have less unhealthy days, therefore better HRQoL, compared to their younger counterparts. This is consistent with Trief et al., who found that elderly adults with diabetes reported better social functioning, using the SF-36 and significantly less diabetes-related emotional distress using the PAID [6]. Sundaram et al. found that older age significantly explained higher MCS-12 scores indicating better mental health status [5]. Our findings are contradictory to the Brown et al. study; they found older adults with diabetes reporting nearly twice as many unhealthy days, but they categorised older adults as >50 years [10]. It can be argued that the unhealthy day's summary measure picked up more general issues around physical and emotional stress which may be higher in the younger age groups, with the general demands of life. Few of our older patients with type 2 diabetes were using insulin. It is possible that some of these patients may have been recently diagnosed with diabetes and may not have progressed to having diabetes complications or to require insulin for good glycaemic control. This suggests that insulin use is a burden on quality of life in diabetes. In the present study, insulin use was significantly associated with reporting unhealthy days. Our findings concur with research using both generic and disease-specific measures; where insulin use was associated with poorer scores on the ADDQoL [3], [4], [5], [12], EQ-5D [3], SF-12, [3], [8] and more unhealthy days per month [10]. Our findings complement the research consensus on the negative association between diabetes complications and QoL [3], [4], [8]. We found significant associations between unhealthy days and increasing number of complications. However, our study did not differentiate between the severities of the complications reported. Future studies should include ranking the severity of complications with HRQoL. We found that having a BMI between 25–29.9 kg/m2 (overweight) was protective of reporting unhealthy days when compared to being a healthy weight or obese. We did not find any association between obesity (BMI>30 kg/m2) and unhealthy days which was has been found in previous work using both generic and disease-specific instruments [3], [5], [8]. It can be hypothesised that obesity can contribute to poor mobility and more psychological issues, including depression, which the unhealthy days summary measure may have detected here but the causal relationship of this cannot be established.

The unhealthy days summary measure in this study and the ADDQoL used in the same population [4] have identified similar determinants of poor QoL. Despite this finding, the Kappa coefficient demonstrated poor agreement between the two measures. Our study is one of the first studies to examine agreement between these two measures, and this finding would suggest that the unhealthy days summary measure and the ADDQoL maybe measuring different constructs. Perhaps this is due to the CDC's HRQoL-4 being derived from the SF-36 which is a measurement of health status, which is a distinctly different construct to QoL, which the ADDQoL measures.

Limitations of the study include the cross-sectional design, which limits causal inference due to uncertainty around the direction of associations. The unhealthy days summary measure ranges from 0–30 days and we may have lost statistical power by collapsing into response categories (one or more vs. none). Our results may not be generalizable to the overall diabetes population, as our study omitted the institutionalised, hospitalised and housebound.

This study enhances current knowledge on determinants of HRQoL in patients with diabetes.

Our findings support and strengthen the hypothesis that people with diabetes are at risk of poor HRQoL; therefore it is an important outcome measure in diabetes management. To the best of our knowledge, the unhealthy day's summary measure has not been used in patients with diabetes outside of the U. S. and has not been assessed for agreement with the ADDQoL instrument. Our study adds to the growing body of literature on the use of the unhealthy days summary measure. Further clinical and community based cross-sectional studies, possibly in tandem with qualitative research, are needed as a means to assess and improve HRQoL in diabetes. With time constraints in clinical practice, the brevity of the unhealthy days summary measure makes it an attractive HRQoL measurement tool as part of routine practice.
